# AI-Driven Three-Dimensional Reconstruction of Fetal Cardiovascular Pathways from Ultrasound Sweep Videos for Congenital Heart Disease Screening

**DOI:** 10.3390/bioengineering13090986

**Published:** 2026-08-26

**Authors:** Naoaki Harada, Masaaki Komatsu, Naoki Teraya, Shohei Wakisaka, Katsuji Takeda, Takashi Natsume, Tomonori Taniguchi, Nobuji Kouno, Satoshi Takahashi, Ken Asada, Syuzo Kaneko, Mayumi Kaneko, Reina Komatsu, Kazuki Iwamoto, Ryu Matsuoka, Akihiko Sekizawa, Ryuji Hamamoto

**Affiliations:** 1 Department of NCC Cancer Science, Biomedical Science and Engineering Track, Graduate School of Medical and Dental Sciences, Institute of Science Tokyo, 1-5-45 Yushima, Bunkyo-ku, Tokyo 113-8510, Japan; naharad@ncc.go.jp (N.H.); katake2@ncc.go.jp (K.T.); 2 Division of Medical AI Research and Development, National Cancer Center Research Institute, 5-1-1 Tsukiji, Chuo-ku, Tokyo 104-0045, Japantanatsum@ncc.go.jp (T.N.); totanig2@ncc.go.jp (T.T.); nkouno@ncc.go.jp (N.K.); sing.monotonyflower@gmail.com (S.T.); ken.asada@riken.jp (K.A.); sykaneko@ncc.go.jp (S.K.); 3 Digital Health Platform Development Office, Healthcare Business Unit, Fujitsu Japan Ltd., 1-5 Omiya-cho, Saiwai-ku, Kawasaki 212-0014, Japan; iwamoto.kazuki@fujitsu.com; 4 AI Medical Engineering Team, RIKEN Center for Advanced Intelligence Project, 1-4-1 Nihonbashi, Chuo-ku, Tokyo 103-0027, Japan; 5 Department of Obstetrics and Gynecology, Showa Medical University School of Medicine, 1-5-8 Hatanodai, Shinagawa-ku, Tokyo 142-8666, Japan; n.teraya@med.showa-u.ac.jp (N.T.); mayumikaneko@med.showa-u.ac.jp (M.K.); rkomatsu@med.showa-u.ac.jp (R.K.); ryu@med.showa-u.ac.jp (R.M.); sekizawa@med.showa-u.ac.jp (A.S.); 6 Department of Obstetrics and Gynecology, Graduate School of Medicine, The University of Tokyo, 7-3-1 Hongo, Bunkyo-ku, Tokyo 113-8655, Japan; 7 Department of Obstetrics and Gynecology, Tohoku University Graduate School of Medicine, 2-1 Seiryomachi, Aoba-ku, Sendai 980-8574, Japan

**Keywords:** fetal cardiac ultrasound screening, three-dimensional visualization, cardiovascular structures, continuity, artificial intelligence

## Abstract

Fetal cardiac ultrasound screening is vital for the prenatal diagnosis of congenital heart disease (CHD). However, understanding complex cardiovascular structures and continuity from two-dimensional (2D) ultrasound images remains challenging. Therefore, this study developed and evaluated an artificial intelligence (AI)-based three-dimensional (3D) visualization approach that reconstructs fetal cardiovascular structures from 2D ultrasound sweep video. To this end, information from the adjacent frames was used. Instead of interpreting each ultrasound frame independently, this method incorporates neighboring frames, enabling the continuous visualization of the right- and left-sided functional circulatory pathways. Our method achieved a mean Dice coefficient of 0.785 on an independent test dataset, demonstrating that the adjacent frame information can provide stable 3D segmentation. Diffusion-based anatomical plausibility analysis trained exclusively on normal anatomies showed exploratory discrimination between normal fetuses and fetuses with CHD (AUC, 0.861; 95% CI, 0.695–0.980). In a clinical comparison study involving 12 obstetricians, the introduction of this AI-driven 3D cardiovascular visualization slightly improved the screening accuracy from 0.779 to 0.808 and significantly improved the confidence-weighted accuracy score (*p* = 0.002). Our approach can potentially support examiners and facilitate fetal cardiac ultrasound screenings.

## 1. Introduction

Fetal ultrasound is commonly used in prenatal care owing to its noninvasive nature, ability to provide real-time imaging, and minimal impact on the mother and fetus [1]. Congenital heart disease (CHD) ranks among the most prevalent congenital anomalies, making precise prenatal diagnosis vital for guiding perinatal care, delivery planning, and postnatal treatments [2,3,4]. A thorough fetal cardiac assessment involves examining several standard cardiac planes, such as the four-chamber view (4CV), left and right ventricular outflow tract views, three-vessel view (3VV), and three-vessel trachea view (3VTV) [5]. However, accurate evaluation remains difficult owing to fetal motion, acoustic shadowing, maternal body habitus, small anatomical features, and high operator dependency [6,7]. Typically, clinicians must mentally reconstruct three-dimensional (3D) cardiovascular anatomy from a series of two-dimensional (2D) ultrasound images, a process that requires significant skills and experience [7,8].

Recent progress in artificial intelligence (AI) has shown promising results in fetal ultrasound analysis, such as the detection of standard planes, segmentation of anatomy, measurement of biometric data, and classification of abnormalities [9,10,11]. These developments aim to reduce inter- and intra-examiner variability, boost the efficiency of ultrasound procedures, and improve the diagnostic accuracy [12,13]. However, most current methods focus on individual 2D images or separate video frames, limiting their ability to interpret complex cardiovascular structures and spatial anatomical relationships. Several congenital cardiovascular abnormalities are characterized by localized structural defects visible on a single plane, abnormal vessel arrangement, and disrupted anatomical continuity [14].

**Figure 1:**
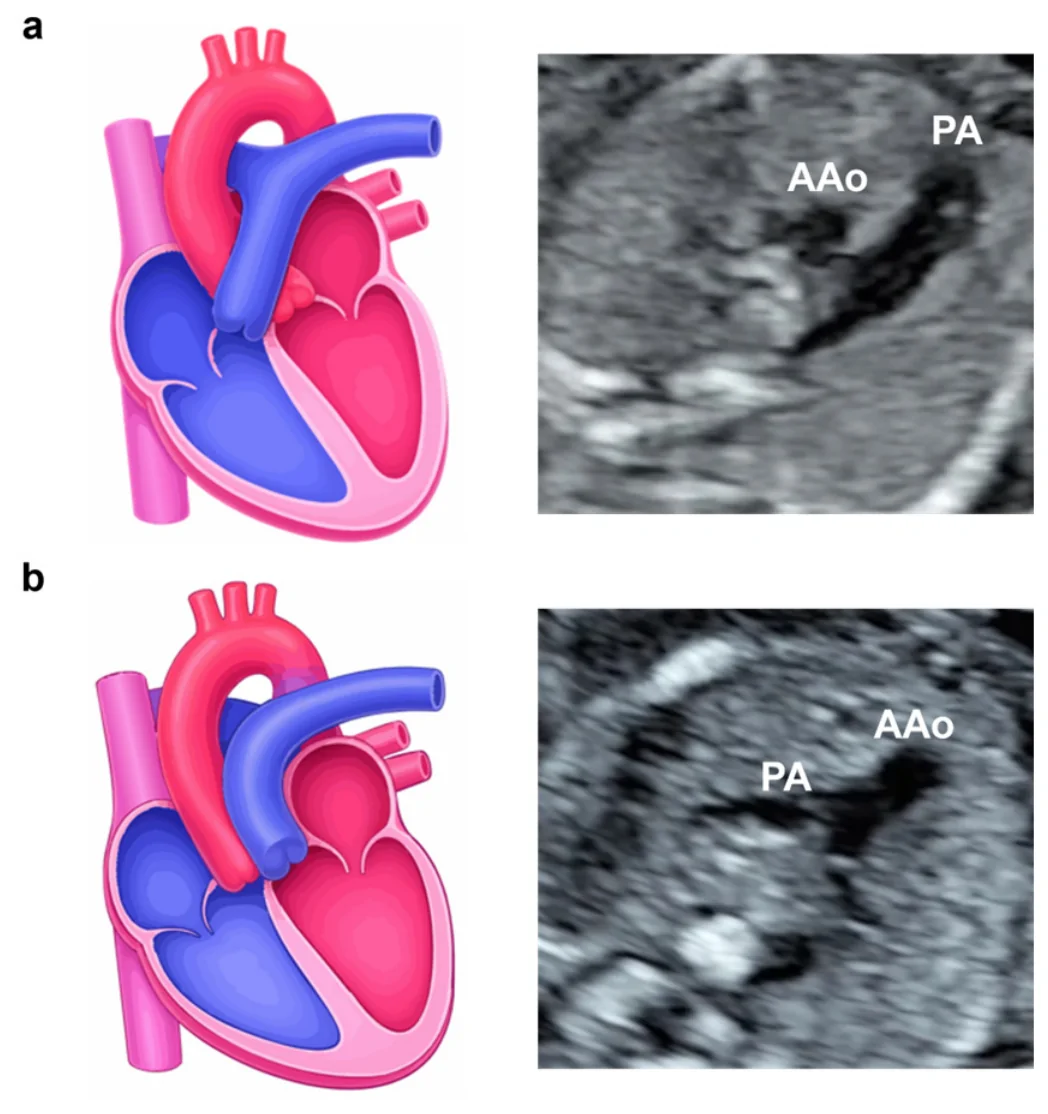
Schematic cardiovascular illustrations and 3VV ultrasound images of a normal fetus (**a**) and a fetus with TGA (**b**). Abnormal connections and parallel orientations of the great vessels are observed in TGA. However, detecting TGA findings using a single 2D image alone, without information on vascular continuity, can be challenging. 3VV, three-vessel view; TGA, transposition of the great arteries; 2D, two-dimensional; PA, pulmonary artery; AAo, ascending aorta.

In prenatal screening, conditions such as transposition of the great arteries (TGA) and total anomalous pulmonary venous connection (TAPVC) should not be overlooked as severe CHDs. In TGA, the aorta arises from the right ventricle and the pulmonary artery arises from the left, resulting in a discrepancy between the ventricles and arteries [15]. One key ultrasound sign of TGA is the I-sign, which is observed on the 3VTV. The aorta is depicted in an “I” shape, originating from the right ventricle in the anterior region and extending posteriorly, merging with the arch of the ductus arteriosus [16,17]. These ultrasound findings rely on the abnormal connection and parallel course of the great vessels, posing a major challenge for interpreters with limited experience and AI models to capture from a single 2D image alone without information on vascular continuity. When comparing a normal fetus and a fetus with TGA, the pulmonary artery and ascending aorta were clearly switched on the 3VV. In this case, simple object detection techniques for the great vessels may overlook TGA (Figure 1). TAPVC is a cardiac malformation in which all pulmonary veins (PVs) directly return to the right atrium or systemic venous system, thereby disrupting the connection between the PV and left atrium (LA). Several biometric parameters of 4CV can be used to detect TAPVC [18,19]. Furthermore, to capture the PV–LA connection, PV and LA 3D visualization and automatic detection in fetal cardiac ultrasound videos have been proposed [20]. Understanding the complex cardiovascular structures and continuity from 2D ultrasound images remains challenging, and existing methods do not adequately reconstruct these structures in three dimensions for reliable screening of CHD. 3D visualization methods that facilitate the understanding of cardiovascular continuity between neighboring frames can aid in the interpretation of anatomy during fetal cardiac ultrasound screening. Commercially available ultrasound equipment supports 3D and four-dimensional (4D) imaging; however, their effective use remains technically demanding for inexperienced examiners and requires substantial expertise [21]. A 3D visualization approach using conventional 2D ultrasound videos obtained during routine clinical practice can address these challenges.

**Figure 2:**
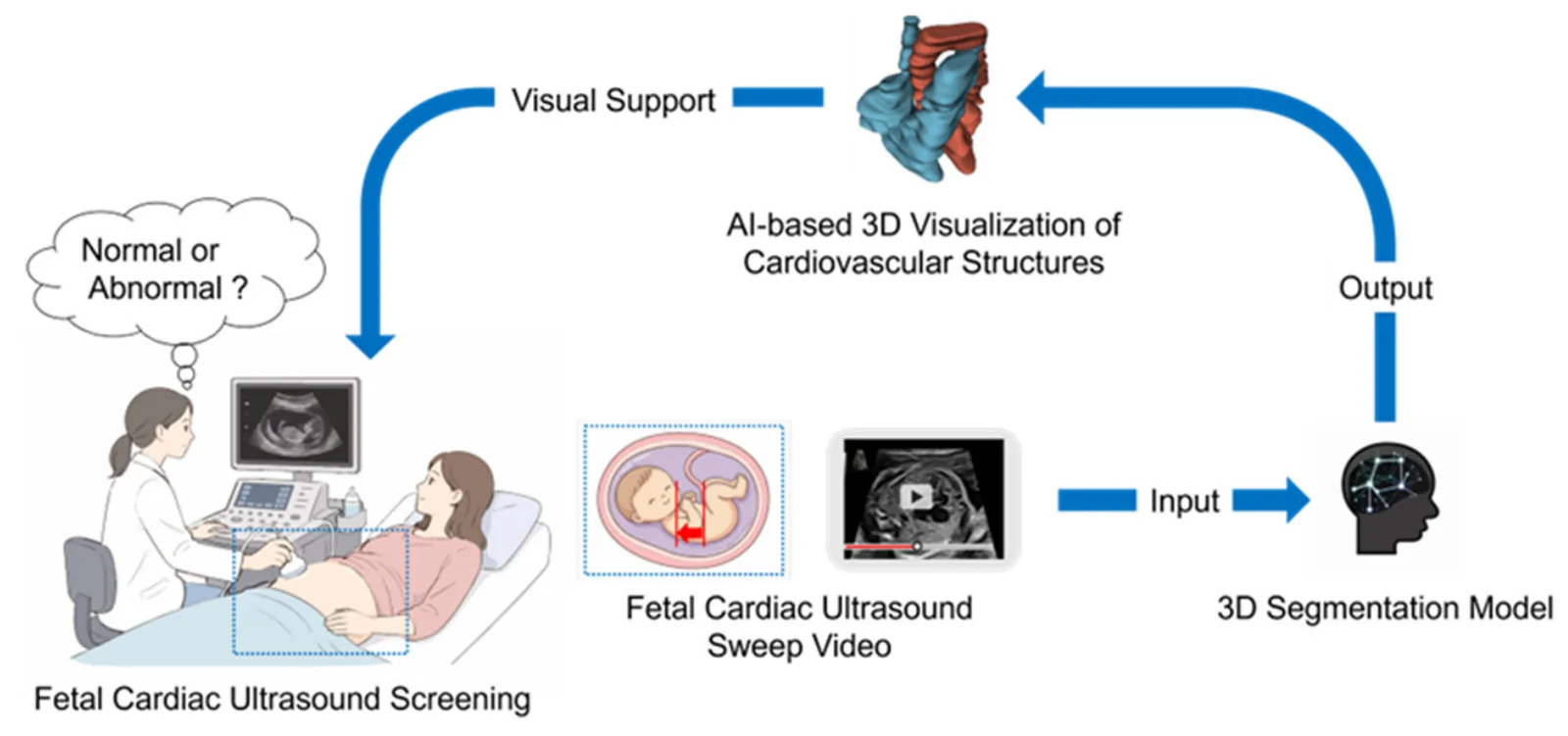
Proposed workflow for fetal cardiac ultrasound screening using AI-driven 3D visual support. AI-driven 3D visualization images of cardiovascular structures are generated from conventional 2D ultrasound videos obtained during routine clinical practice. The red arrows indicate the probe-sweep direction in fetal cardiac ultrasound screening, and the blue arrows show the workflow and data flow. AI, artificial intelligence; 2D, two-dimensional; 3D, three-dimensional.

In this study, we developed an AI-driven framework for generating pseudo-3D representations of fetal cardiovascular pathways from routine ultrasound sweep videos (Figure 2). Rather than introducing an entirely new segmentation backbone, the principal methodological contribution of this work lies in adapting volumetric segmentation to freehand sweep data by explicitly modeling sweep-sequential inter-slice relationships and incorporating pathway-level representation learning for the right- and left-sided circulatory pathways. Specifically, the framework integrates a 3D encoder, a sweep–spatial fusion module, and anatomy-aware contrastive regularization to capture complementary information across successive sweep frames while preserving anatomically meaningful pathway representations. We further evaluated whether the resulting pseudo-3D representations retained clinically relevant cardiovascular morphology through anatomical plausibility analysis and a clinical comparison study.

## 2. Materials and Methods

### 2.1. Dataset

Fetal cardiac ultrasound examinations were retrospectively collected at Showa Medical University Hospitals between 2018 and 2025. The dataset consisted of 197 videos from 160 normal fetuses and 6 videos from 6 fetuses with CHD, acquired using Voluson^®^ E10 (GE Healthcare, Chicago, IL, USA). The gestational age ranged from 18 to 34 weeks. Each examination included a freehand sweep covering the standard 4CV, 3VV, and 3VTV. The acquisition duration was approximately 10–15 s. A total of 39 videos from 30 normal fetuses were reserved as an independent held-out test set, whereas the remaining 158 videos from 130 normal fetuses were used for five-fold cross-validation. All dataset partitioning was performed at the fetus level. When multiple sweep videos were available from the same fetus, all videos from that fetus were assigned exclusively to the same subset, thereby preventing subject-level information leakage across the training, validation, and test sets. In each cross-validation fold, 104 fetuses were assigned to the training subset and 26 fetuses to the validation subset. Regarding the six CHD cases, this study focused on CHDs accompanied by vascular anatomical abnormalities. The diagnoses included TGA, double-outlet right ventricle (DORV) + ventricular septal defect (VSD), DORV + VSD + persistent left superior vena cava, DORV + TGA + coarctation of the aorta (CoA), DORV + TGA + VSD + CoA, and right aortic arch.

Ultrasound sweep videos were divided into separate image frames. Annotation was performed manually by experienced fellows and reviewed by board-certified fetal echocardiography experts. To reduce the annotation burden, labels were generated every four frames throughout the entire sweep sequence. Three semantic classes were defined: background, right-sided cardiac structures, and left-sided cardiac structures. The right-sided cardiac structures included the right atrium, right ventricle, pulmonary artery, and superior vena cava. The left-sided cardiac structures included the LA, left ventricle, ascending aorta, aortic arch, and descending aorta.

### 2.2. Data Preprocessing and 3D Visualization

Unlike spatiotemporal image correlation (STIC) [22], the proposed framework uses freehand ultrasound sweep videos without cardiac synchronization or electrocardiographic gating. Consecutive frames extracted from each sweep sequence were stacked along the sweep trajectory to construct a pseudo-volumetric representation. The sweep direction was treated as the depth axis, transforming sequential 2D ultrasound images into a pseudo-3D volume. Because probe position and sweep velocity were not externally tracked, the inter-frame spacing along the sweep axis could not be calibrated to physical distance. Accordingly, the sweep axis represents the sequential order of the acquired frames rather than a metrically calibrated spatial coordinate. The reconstructed volume should therefore be interpreted as a pseudo-3D anatomical representation rather than a geometrically calibrated 3D volume. In addition, because the ultrasound sweeps were acquired without cardiac synchronization or ECG gating, consecutive frames may correspond to different phases of the cardiac cycle. Consequently, inter-frame anatomical changes may reflect not only probe displacement but also cardiac motion. All volumes were reoriented and resized to 96 × 128 × 160 voxels (depth × height × width). Trilinear interpolation was applied to the intensity images, whereas nearest-neighbor interpolation was used for the segmentation labels. The intensity values were normalized from [0, 255] to [−1, 1]. During training, 96 × 96 × 96 voxel patches were sampled using class-balanced positive–negative sampling. Data augmentation included random Gaussian noise, bias field perturbation, intensity scaling, intensity shifting, random flipping, random zooming, and 3D elastic deformation.

### 2.3. Training and Evaluation of 3D Segmentation Models

Representative 3D medical image segmentation models, including SegResNet [23], SwinUNETR-v2 [24,25], MedNeXt [26], SwinMM [27], and 3DINO [28], were compared with the proposed model. These five competing state-of-the-art models were selected to represent convolutional neural networks, transformer-based architectures, modern ConvNeXt-style volumetric networks, self-supervised multi-view learning frameworks, and large-scale self-supervised foundation models. All baseline models were trained and evaluated using the same training, validation, and test splits, preprocessing pipeline, optimization settings, and evaluation metrics as those of our proposed model to ensure a fair comparison. The network was optimized using AdamW with an initial learning rate of 1 × 10^−3^ and weight decay of 1 × 10^−4^. Cosine annealing scheduling was used with a minimum learning rate of 1 × 10^−5^.

The models were trained for up to 500 epochs with early stopping. Five models were independently trained using the five cross-validation training–validation splits. For evaluation on the independent held-out test set, each model generated voxel-wise logits, which were averaged across the five models before final class assignment. The ensemble performance reported in the main analysis was calculated from these logit-averaged predictions. In addition, each fold-specific model was evaluated separately on the same held-out test set to characterize between-model variability. Fold-specific performance was summarized descriptively as the mean ± standard deviation across the five independently trained models; these values were intended to reflect between-model variability and were not interpreted as confidence intervals. Segmentation performance was assessed using the mean values of the Dice coefficient (mDice) [29], surface Dice (msDice) [30], and the 95% Hausdorff distance (mHD95) [31], averaged across the right- and left-sided cardiovascular structures. To further assess structural continuity, we calculated the mean centerline Dice coefficient (mclDice), mean connected-components absolute error (mCC error), and mean skeleton-continuity absolute error (mSC error). CC error was defined as the absolute difference in the number of connected components between the predicted and reference segmentations. Skeleton continuity was defined as the proportion of skeleton voxels belonging to the largest connected skeleton component, and SC error was calculated as the absolute difference in this proportion between the predicted and reference segmentations.

All test-set metrics were summarized at the fetus level. When multiple videos were available from the same fetus, video-level metric values were first averaged within each fetus. For each metric, values for the right- and left-sided cardiovascular structures were subsequently averaged to obtain a single fetus-level value. Ninety-five percent confidence intervals (CIs) for the mean performance of each model and for paired differences between the proposed model and each comparator were estimated using 10,000 fetus-level bootstrap resamples. Pairwise comparisons were performed using the Wilcoxon signed-rank test on fetus-level paired observations. To account for multiple comparisons, *p* values were adjusted using the Holm method separately within each metric across the five comparator models. Rank-biserial correlation was additionally reported as the effect size. Its sign followed the direction of the paired difference (proposed model − comparator); therefore, for distance- and error-based metrics, negative values indicated lower—and thus more favorable—values for the proposed model.

### 2.4. Our Proposed Model

**Figure 3:**
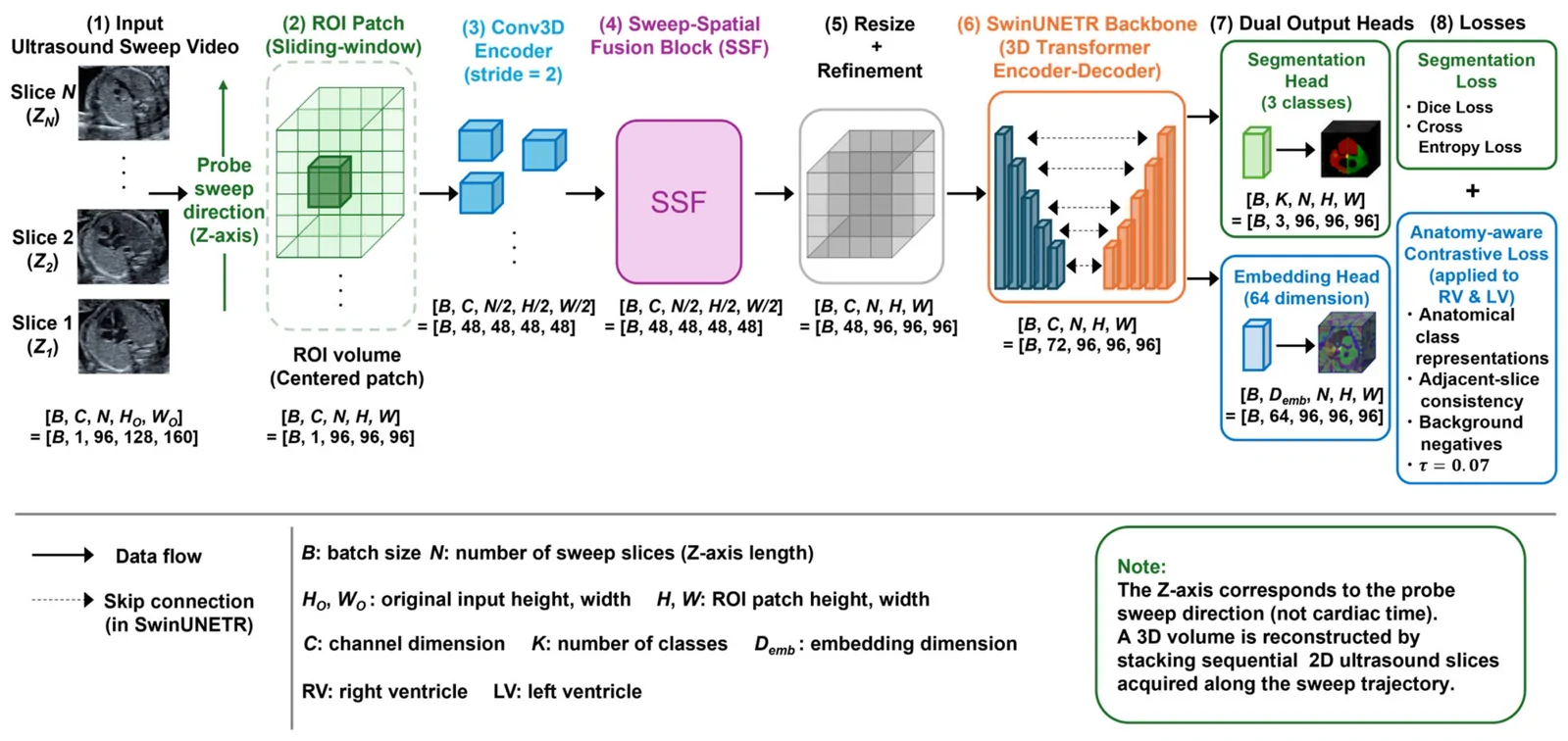
Overall architecture of the proposed 3D segmentation framework. Sequential 2D fetal cardiac ultrasound images acquired along the probe-sweep direction were stacked to create a pseudo-volumetric input. A sliding window strategy was used to extract 3D region-of-interest patches, which were initially processed using a Conv3D encoder with a stride of 2. The resulting feature volume passed through the sweep–spatial fusion block to integrate the inter-slice sequential information along the sweep direction with the in-plane spatial information. The fused features were subsequently resized and refined before being provided to the SwinUNETR-based 3D encoder–decoder backbone. Two output heads were attached to the backbone: a segmentation head that generated voxel-wise predictions for three classes and 64-dimensional embedding head used for auxiliary representation learning. The model was optimized using Dice loss and cross-entropy loss for segmentation, together with an anatomy-aware contrastive loss applied to the right- and left-sided cardiovascular representations. In this framework, the *Z*-axis represents the probe-sweep direction rather than the cardiac time. 2D, two-dimensional; 3D, three-dimensional.

We implemented a SwinUNETR-based segmentation model adapted to pseudo-volumetric fetal cardiac ultrasound data. The architecture consisted of a shallow Conv3D encoder, sweep–spatial fusion (SSF) block, SwinUNETR backbone, and dual output heads (Figure 3). The segmentation head generated voxel-wise predictions, whereas the embedding head generated 64-dimensional feature embeddings used for auxiliary contrastive regularization.

**Figure 4:**
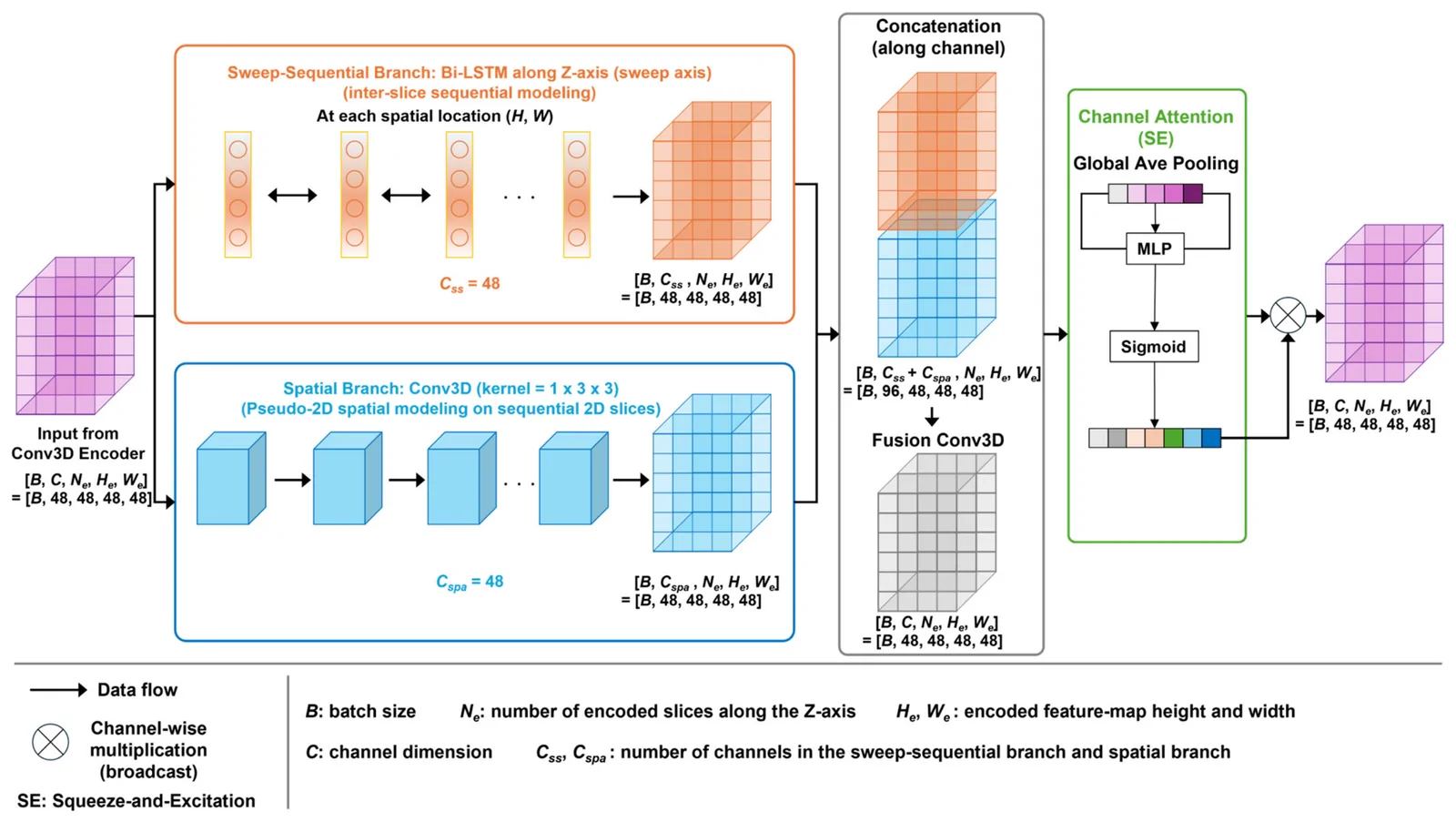
Architecture of sweep–spatial fusion block. The feature volume generated by the Conv3D encoder was processed in two parallel branches. In the sweep-sequential branch, bidirectional long short-term memory networks were applied along the sweep axis at each spatial location to model sequential inter-slice dependencies. In the spatial branch, a Conv3D layer with a kernel size of 1 × 3 × 3 was used to extract the in-plane spatial features independently for each slice. The outputs of the sweep-sequential and spatial branches were concatenated along the channel dimension and subsequently refined using a squeeze-and-excitation channel-attention module. The resulting channel weights were applied to the concatenated feature volume by channel-wise multiplication to generate the output of the sweep–spatial fusion block.

An SSF block was introduced to integrate sweep-sequential inter-slice dependencies with intra-slice anatomical features along the probe-sweep trajectory (Figure 4). The input feature map generated using the 3D convolutional encoder is represented by Equation (1).

(1) $$F\;\in \;R^{\left(C\times D\times H\times W\right)},$$

where C, D, H, and W represent the channel, sweep-axis, height, and width dimensions, respectively. The SSF block consisted of sweep-sequential and spatial branches. As formulated in Equations (2) and (3), the sweep-sequential branch models the inter-slice dependencies along the sweep axis using a bidirectional long short-term memory network, whereas the spatial branch extracts the intra-slice anatomical features using a 3D convolution with a kernel size of 1×3×3.

(2) $$F_{ss}=BiLSTM\left(F\right),F_{ss}\;\in \;R^{\left(C_{ss}\times D\times H\times W\right)},\;C_{ss}=48$$

(3) $$F_{spa}={Conv}_{1\times 3\times 3}\left(F\right),\;F_{spa}\;\in \;R^{\left(C_{spa}\times D\times H\times W\right)},\;C_{spa}=48$$

The sweep-sequential and spatial feature maps were concatenated along the channel dimension, as shown in Equation (4).

(4) $$F_{cat}=\left[F_{ss};\;F_{spa}\right]\;\in \;R^{\left(C_{ss}+C_{spa}\right)\times D\times H\times W}=R^{\left(96\times D\times H\times W\right)}$$

Channel-wise recalibration was subsequently performed using a squeeze-and-excitation mechanism, as expressed in Equation (5).

(5) $$\alpha =\sigma (W_{2}\;\delta (W_{1}\;GAP(F_{cat})))$$

The operators GAP, δ, and σ correspond to global average pooling, GELU activation, and sigmoid activation, respectively. Element-wise multiplication of the channel attention weights α and the concatenated feature map $F_{cat}$ yielded the final SSF representation, which was expressed in Equation (6).

(6) $$F_{SSF}=\alpha ⊙F_{cat}$$

The fused feature representation was forwarded to the SwinUNETR encoder–decoder architecture. Hierarchical transformer blocks enable the simultaneous modeling of local anatomical structures and long-range contextual information. Skip connections were used between the encoder and decoder stages to maintain spatial information.

We incorporated an auxiliary anatomy-aware contrastive loss (ACL) to promote feature consistency between adjacent slices. An embedding head generated a 64-dimensional embedding map $E\;=R^{\left(64\times D\times H\times W\right)}$. For anatomical class c in slice d, the corresponding class-level representation was computed by averaging the voxel embeddings within the segmentation region $M_{c}^{d}$, as defined in Equation (7).

(7) $$r_{c}^{d}=\frac{1}{\left|M_{c}^{d}\right|}\sum_{i\;\in \;M_{c}^{d}}E_{i}$$

where $M_{c}^{d}$ represents the set of voxels belonging to class c in the ground-truth segmentation mask at slice d. Before prototype computation, one iteration of two-dimensional erosion was applied independently to each slice to reduce the influence of boundary voxels. To avoid forming positive pairs when the corresponding cardiovascular pathway was absent or only minimally represented, representations of the same functional circulatory pathway in adjacent slices, $\left(r_{c}^{d},r_{c}^{d+1}\right)$, were treated as positive pairs only when the corresponding class contained at least four voxels in both eroded masks. Thus, the ACL did not assume voxel-wise correspondence or that adjacent slices depicted exactly the same local anatomical structure; rather, it encouraged representation consistency of the same functional circulatory pathway across successive slices in which that pathway was sufficiently represented. For each valid anchor representation of class c at slice d, the representation of the same class at slice d+1 was used as the positive sample, whereas the representations of the opposite circulatory class and the background at slice d+1 were used as negative samples. Accordingly, each valid anchor was contrasted with one positive and two negative class-level representations. The ACL was formulated using the InfoNCE objective in Equation (8).

(8) $$L_{ACL}=-\sum_{c,d}\log\frac{exp\left(sim\left(r_{c}^{d},\;\;r_{c}^{d+1}\right)/\tau \right)}{\sum_{k}exp\left(sim\left(r_{c}^{d},\;\;r_{k}^{d+1}\right)/\tau \right)}$$

where τ represents the temperature parameter and was set to 0.07. Cosine similarity between two representations a and b was defined as $sim\left(a,\;b\right)=\;\frac{a^{T}b}{\left\|a\right\|\left\|b\right\|}$. The final training objective combined the Dice loss, cross-entropy loss, and the proposed ACL, as shown in Equation (9).

(9) $$L=L_{Dice}+L_{CE}+\lambda L_{ACL}$$

where λ denotes the weighting coefficient for the ACL and was set to 1.0.

To assess the contributions of the two principal methodological additions to the SwinUNETR backbone, we performed a module-level ablation analysis using four model configurations: SwinUNETR alone, SwinUNETR with anatomy-aware contrastive loss (ACL), the sweep–spatial fusion (SSF)-based architecture without ACL, and the complete SSF-based architecture with ACL (Ours). The SSF-based architecture comprised a shallow Conv3D encoder and the SSF block, which together formed an integrated sweep-aware front end. In the present ablation analysis, SSF was evaluated as a single methodological unit because the analysis was designed to assess the contributions of the two principal methodological additions to the SwinUNETR backbone, namely SSF and ACL, rather than to isolate the effects of individual components within the SSF module. The BiLSTM branch and SE attention were therefore treated as complementary components of a unified fusion mechanism. All configurations were evaluated on the same independent held-out test set using five-model logit-averaged ensembles. Performance was summarized at the fetus level using the same six segmentation and continuity metrics described above, and 95% confidence intervals were estimated using 10,000 fetus-level bootstrap resamples. Prespecified paired comparisons were performed using the Wilcoxon signed-rank test, with Holm correction applied separately within each metric across the four module-level contrasts.

### 2.5. Diffusion-Based Anatomical Plausibility Assessment

A preliminary assessment was conducted before the clinical comparison study to determine whether the outputs of our 3D segmentation model could exhibit visually recognizable morphological differences between normal fetuses and fetuses with CHD. As the segmentation model was trained exclusively on normal fetuses, we quantitatively determined whether its predictions for fetuses with CHD deviated from the distribution of normal fetal cardiovascular anatomies. Therefore, as an exploratory analysis, we conducted a reconstruction-based anatomical plausibility assessment using a Denoising Diffusion Probabilistic Model (DDPM). This analysis was designed to evaluate whether the reconstructed representations retained deviations from normal cardiovascular morphology and was not intended to establish a clinically validated CHD diagnostic model. DDPMs can learn the distribution of normal anatomy and identify deviations from the learned normal patterns through reconstruction-based anomaly analysis [32,33,34,35].

The DDPM was trained using 104 normal fetuses and validated using 26 additional normal fetuses. The training data comprised manually annotated segmentation masks used in the segmentation task, represented as 3D label volumes with two anatomical classes corresponding to the right- and left-sided cardiovascular structures. By training exclusively on normal fetuses, the model was expected to capture the distribution of normal cardiovascular anatomies. During inference, the predicted 3D segmentation volumes produced by the segmentation model were used as the inputs. The input volume was denoted by ${{\hat{x}}_{0}}^{\left(m\right)}$. For each input volume x_0_, Gaussian noise was added during each Monte Carlo iteration m at a fixed diffusion timestep of t = 850, corresponding to 85% of the total diffusion process (T = 1000). The diffusion timestep was selected based on visual inspection of the forward-noising process; at this noise level, the original vascular trajectories became difficult to visually recognize. The trained DDPM was used to estimate the corresponding original volume, denoted by ${{\hat{x}}_{0}}^{\left(m\right)}$. $A^{\left(m\right)}$, the anomaly map for each iteration, was defined as the voxel-wise absolute difference between the input and reconstructed volumes, as expressed in Equation (10) (Supplementary Figure S1).

(10) $$A^{\left(m\right)}=\left|x_{0}-{{\hat{x}}_{0}}^{\left(m\right)}\right|$$

The final anomaly map A was obtained by averaging the anomaly maps from 20 Monte Carlo inference runs, as expressed in Equation (11), with M set to 20. Twenty Monte Carlo iterations were used to reduce stochastic variability arising from the diffusion noise while maintaining computational feasibility.

(11) $$A=\frac{1}{M}\sum_{m=1}^{M}A^{\left(m\right)},\;M=20$$

After Gaussian smoothing of the anomaly map, the anomaly score for each fetus was defined as the mean anomaly value of the 10 voxels with the highest anomaly values. Receiver operating characteristic (ROC) analysis and the area under the ROC curve (AUC) were used to evaluate the discrimination between normal fetuses and fetuses with CHD. To assess algorithmic robustness to the stochastic noise realization, the complete inference and evaluation procedure was repeated using five different random seeds. To assess the sensitivity of the anomaly score to the number of highest-anomaly voxels, the ROC analysis was additionally repeated using top-k values of 1, 5, 20, 50, and 100 voxels, with the top-10 setting retained as the primary analysis. This sensitivity analysis was performed for each of the same five random seeds. Ninety-five percent CIs for the ROC AUC were estimated using 10,000 fetus-level bootstrap resamples for each random seed and top-k setting. Sensitivity and specificity were calculated at the optimal thresholds determined using the Youden index. The data in the final column are reported as the mean ± standard deviation across the five random seeds. The ROC curve obtained using a random seed of 300 is shown as a representative example in Supplementary Figure S2.

### 2.6. Design of a Clinical Comparison Study

A clinical comparison study was conducted to evaluate whether 3D cardiovascular visualizations generated by the proposed model could support the screening performance of examiners. Twelve obstetricians participated in the study: five experts certified in fetal echocardiography and seven fellows with experience in fetal cardiac ultrasound screening. The test dataset included 20 fetal cardiac ultrasound sweep videos, consisting of 14 normal fetuses and 6 fetuses with CHD. All participants first evaluated the same 20 cases using the ultrasound videos alone and subsequently reevaluated the same cases with AI-driven 3D visualization. The order of case presentation was randomized in each reading session. A washout interval of at least 5 min was required between the two reading sessions to reduce recall bias, although participants were permitted to take a longer interval before starting the second session. Each participant was given 20 randomly numbered videos and determined whether they were normal or abnormal with five confidence levels (integers of 1–5). A higher number indicates higher confidence. The screening accuracy and confidence-weighted accuracy scores were calculated and compared between those with and without AI-driven 3D cardiovascular visualization. Each examiner was treated as the statistical unit. For each reading condition, accuracy and confidence-weighted accuracy were first averaged across the 20 cases for each examiner, yielding one examiner-level value per outcome and condition. Ninety-five percent confidence intervals (CIs) for the mean outcome under each condition and for the paired mean difference between conditions were estimated using 10,000 examiner-level bootstrap resamples. Paired comparisons between the video-only and video-plus-3D conditions were performed using the two-sided Wilcoxon signed-rank test. Rank-biserial correlation was additionally reported as the effect size, with positive values indicating higher scores in the video-plus-3D condition.

### 2.7. Software and Computational Environment

For segmentation model development and evaluation, the proposed model and all comparison models except SwinMM and 3DINO were implemented in Python 3.10.17 using PyTorch 2.8.0, MONAI 1.5.1, NumPy 1.26.4, SciPy 1.15.3, and scikit-image 0.24.0. SwinMM was implemented and evaluated in a separate environment comprising Python 3.10.17, PyTorch 2.10.0, MONAI 1.4.0, NumPy 1.24.4, SciPy 1.11.1, and scikit-image 0.24.0. 3DINO was implemented and evaluated using Python 3.11.13, PyTorch 2.9.1, MONAI 1.5.1, NumPy 2.4.6, SciPy 1.17.1, and scikit-image 0.26.0.

For anomaly detection, the DDPM-based model was implemented and evaluated using Python 3.11.13, PyTorch 2.9.0, MONAI 1.5.1, NumPy 2.3.4, SciPy 1.16.3, and scikit-image 0.25.2.

GPU-accelerated experiments were performed using CUDA 12.8, except for 3DINO, for which CUDA 12.6 was used. cuDNN 9.10.2 was used across all GPU environments.

For the clinical comparison study, study-set preparation and statistical analyses were performed using Python 3.10.10. openpyxl 3.1.1 and OpenCV 4.7.0 were used for study-set preparation and video processing, respectively. Statistical analyses were conducted using NumPy 1.24.2, pandas 1.5.3, SciPy 1.10.1, scikit-learn 1.2.2, and statsmodels 0.13.5. Matplotlib 3.7.1 was used for data visualization.

## 3. Results

### 3.1. Segmentation Performance

Table 1 summarizes fetus-level segmentation and continuity performance for the proposed model and the comparator models. The proposed model achieved the highest mDice among all evaluated models. Its msDice and mclDice were comparable to those of MedNeXt, which yielded the highest values for these two metrics. MedNeXt achieved the lowest mCC error, SwinUNETR-v2 the lowest mSC error, and 3DINO the lowest mHD95. Thus, no single model consistently outperformed the others across all overlap-, surface-, and continuity-oriented metrics. Fetus-level paired statistical comparisons of the ensemble predictions are presented in Supplementary Table S1. After Holm correction, no statistically significant differences were observed between the proposed model and either MedNeXt or SwinUNETR-v2 across the evaluated metrics. In contrast, statistically significant differences were observed between the proposed model and SegResNet, SwinMM, or 3DINO for one or more metrics. Fold-specific single-model performance and the corresponding five-model ensemble results are summarized in Supplementary Table S2. For the proposed model, the mean mDice across the five independently trained fold-specific models was 0.769 ± 0.002, whereas the five-model logit-averaged ensemble achieved an mDice of 0.785. The corresponding msDice values were 0.843 ± 0.003 and 0.859, respectively.

A module-level ablation analysis was additionally performed to assess the contributions of the SSF-based architecture and ACL (Supplementary Table S3). Compared with the SwinUNETR baseline, the SSF-only configuration showed modest numerical improvements in several metrics, including an increase in mDice from 0.782 to 0.786 and in mclDice from 0.854 to 0.857, together with a reduction in mSC error from 0.123 to 0.113. The ACL-only configuration likewise showed small numerical improvements over the baseline, with slightly higher mDice and msDice and a slightly lower mHD95. However, the complete model did not consistently outperform the partial configurations across all evaluated metrics. It achieved the lowest mCC error (1.892), whereas the SSF-only configuration achieved the highest mDice and mclDice and the lowest mSC error. None of the prespecified module-level contrasts remained statistically significant after Holm correction. Overall, these findings suggest that the effects of SSF and ACL were modest and metric dependent rather than consistently additive across performance measures.

### 3.2. Qualitative 3D Visualization

**Figure 5:**
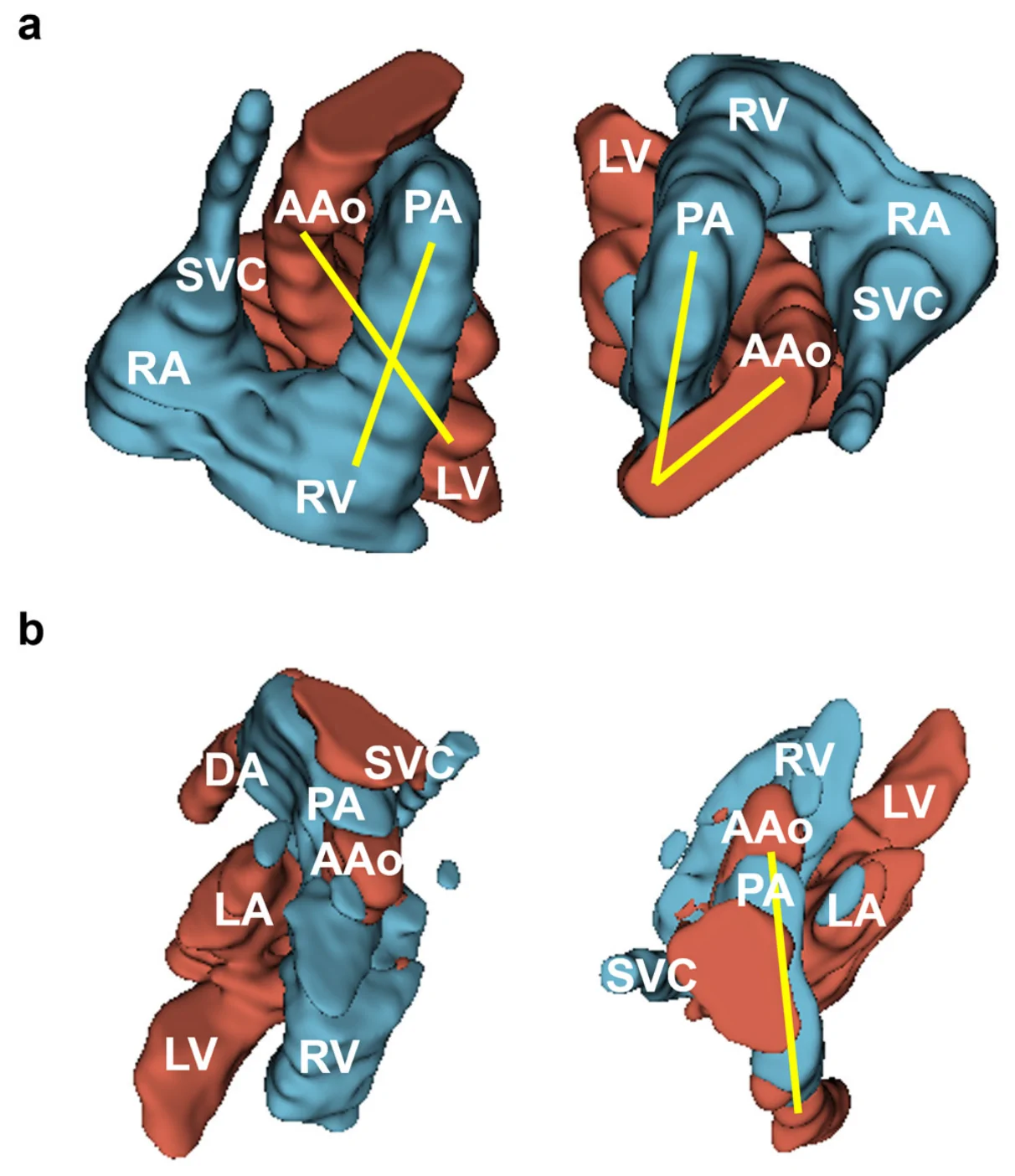
Representative 3D visualization images of a normal fetus (**a**) and a TGA-type DORV case (**b**). Crossings of the great vessels were observed in the outflow tract, and the V-sign was confirmed on the 3VTV in normal fetuses. In contrast, the great vessels originated from the right ventricle, and no crossing was observed in the outflow tract, and the I-sign was confirmed on the 3VTV in this fetus with TGA-type DORV. The yellow lines highlight the characteristic spatial course of the great vessels, including the crossing configuration and V-shaped sign in the normal fetus (**a**) and the I-shaped sign in the TGA-type DORV case (**b**). TGA, transposition of the great arteries; DORV, double-outlet right ventricle; 3VTV, three-vessel trachea view; AAo, ascending aorta; PA, pulmonary artery; RV, right ventricle; LV, left ventricle; RA, right atrium; LA, left atrium; SVC, superior vena cava; DA, descending aorta; 3D, three-dimensional.

Representative 3D visualization examples are illustrated in Figure 5. Our model successfully reconstructed anatomically plausible 3D cardiovascular structures from 2D ultrasound sweep video. In normal fetuses, the major cardiac chambers and great vessels were reconstructed with continuous spatial relationships; conversely, in fetuses with CHD, characteristic anatomical abnormalities were preserved in the reconstructed volumes, enabling intuitive visualization of cardiovascular morphology. The proposed model generated anatomically coherent cardiovascular structures with visually preserved continuity across the sweep direction.

### 3.3. Anatomical Plausibility

A diffusion-based anatomical plausibility analysis was performed using a DDPM trained exclusively on normal cardiovascular anatomy. When applied to the generated 3D visualization results, the anomaly score showed exploratory discrimination between 30 normal fetuses and 6 fetuses with CHDs characterized by vascular anatomical abnormalities, with an AUC of 0.861 (95% CI, 0.695–0.980). Across the five random seeds, the ROC AUC ranged from 0.789 to 0.861, with a mean ± SD of 0.814 ± 0.028, indicating generally similar performance across different stochastic noise realizations (Supplementary Table S4). Sensitivity analysis showed that discrimination performance varied according to the number of highest-anomaly voxels used to calculate the anomaly score. Across the five random seeds, the mean ROC AUC ranged from 0.811 ± 0.023 using the single highest-anomaly voxel to 0.838 ± 0.030 using the 100 highest-anomaly voxels, compared with 0.814 ± 0.028 for the primary top-10 setting. The mean ROC AUC across the five random seeds was 0.811 or higher for all tested top-k settings, whereas individual seed-level AUCs ranged from 0.783 to 0.867 (Supplementary Table S4). These findings indicate that, although the absolute ROC AUC varied modestly according to the random seed and top-k definition, exploratory discrimination between normal and abnormal cardiovascular representations was generally maintained across the evaluated settings. In contrast, the resulting AUC was 0.611 (95% CI, 0.293–0.879) when the same framework was trained using the original fetal cardiac ultrasound sweep videos as input (Supplementary Figure S2a). The AUC was numerically higher for the 3D visualization than for the raw ultrasound videos; however, this was an exploratory comparison.

Furthermore, when conducting the same analysis on another test dataset consisting of 14 normal fetuses and 6 fetuses with CHD in the clinical comparison study, the AUCs were 0.857 (95% CI, 0.653–1.000) for 3D visualization and 0.369 (95% CI, 0.120–0.656) for raw ultrasound videos (Supplementary Figure S2b). A similar numerical pattern was observed in the clinical comparison study cohort, in which the AUC was higher for the 3D visualization than for the raw ultrasound videos. Although these exploratory findings suggest that the generated 3D visualization retained morphological differences between normal and abnormal cardiovascular structures, the small and heterogeneous CHD cohort precludes conclusions regarding diagnostic performance for individual CHD types.

### 3.4. Clinical Comparison Study

Table 2 summarizes the results in the clinical comparison study. Across all 12 examiners, mean screening accuracy increased from 0.779 to 0.808 following the AI-driven 3D visual support, although this difference was not statistically significant. Furthermore, the confidence-weighted accuracy score significantly improved from 6.525 to 6.925 (*p* = 0.002). In exploratory subgroup analyses, confidence-weighted accuracy increased in both experts and fellows, with the increase reaching statistical significance among fellows but not among experts. Given the small numbers of examiners in each subgroup, these subgroups findings should be interpreted cautiously. These findings suggest that AI-driven 3D cardiovascular visualization was associated with a modest increase in screening accuracy and improved confidence-weighted scores across examiners with different levels of fetal cardiac ultrasound experience. Detailed examiner-level statistical results, including 95% CIs for paired differences and rank-biserial correlations, are provided in Supplementary Table S5.

## 4. Discussion

Fetal cardiac ultrasound screening for CHDs remains prevalent; however, ultrasound image acquisition and interpretation require substantial expertise [36,37]. To support fetal echocardiography examiners, several AI-driven software programs have been proposed, some of which use object detection of multiple standard cardiac planes or cardiac substructures [38,39,40]. Recent studies on AI-assisted fetal cardiac ultrasound screening have primarily focused on standard plane detection, view classification, and automated biometric measurements. However, most of these approaches analyze individual planes or static images, and few have investigated the 3D visualization of fetal cardiovascular anatomy and its clinical utility. The fetal cardiovascular system consists of complex 3D structures, making it difficult to understand the spatial relationships between the cardiac chambers and great vessels from 2D ultrasound images. Regarding CHDs with abnormal vessel arrangement and anatomical continuity, such as TGA, it is vital to capture information about vascular continuity.

Herein, we implemented and evaluated an AI-driven 3D visualization of fetal cardiovascular structures using routine fetal cardiac ultrasound sweep videos. The purpose of this study was not to achieve highly detailed anatomical segmentation of each cardiovascular component but to determine whether a simplified, functionally meaningful representation could support clinical interpretation. A clinically oriented aspect of this study was the reconstruction of cardiovascular anatomy as right- and left-sided functional circulatory pathways. In fetal cardiac assessment, along with evaluating individual anatomical components, understanding the continuity and connectivity of cardiovascular structures is often important. Abnormal courses of the great vessels and imbalances between the right- and left-sided circulatory systems are important diagnostic findings in various forms of CHD. Therefore, a unified 3D representation of the circulatory pathways may help examiners intuitively recognize the global cardiovascular anatomy.

STIC, one of the most widely used 3D ultrasound techniques in fetal cardiology, requires volume acquisition over several seconds, during which fetal movement and acoustic shadowing may affect the quality of the reconstructed volumes. Moreover, it mainly provides a 3D visualization of the ultrasound intensity information. In contrast, the proposed framework reconstructs cardiovascular structures using anatomical knowledge learned from expert-annotated 2D ultrasound images. Thus, our approach should not be regarded as a replacement for established 3D ultrasound techniques but as a complementary method that facilitates anatomical interpretation in screening settings. A direct head-to-head comparison with STIC was not performed because paired STIC acquisitions were unavailable in this retrospective dataset. Accordingly, the present study cannot establish superiority over STIC or other dedicated 3D/4D ultrasound techniques with respect to geometric accuracy, image quality, acquisition efficiency, or robustness to motion. Prospective studies using paired acquisitions will therefore be required to enable direct comparison with these established techniques.

No single segmentation architecture consistently outperformed the others across all evaluated metrics, and the proposed model demonstrated competitive overall performance across volumetric overlap, surface agreement, and continuity-oriented measures. Fetus-level paired statistical comparisons of the ensemble predictions are provided in Supplementary Table S1. After Holm correction, the proposed model showed significantly higher mDice than SegResNet, SwinMM, and 3DINO; significantly higher msDice than SwinMM and 3DINO; and significantly higher mclDice than SwinMM and 3DINO. It also showed a significantly lower mHD95 than SwinMM but a significantly higher mHD95 than 3DINO, as well as a significantly lower mCC error than 3DINO. No significant between-model differences in mSC error remained after correction for multiple comparisons. Importantly, no significant differences were observed between the proposed model and either MedNeXt or SwinUNETR-v2 across the evaluated metrics after Holm correction. These findings indicate that the proposed model should be regarded as competitive rather than uniformly superior to existing architectures.

The module-level ablation analysis further suggested that the contributions of the proposed components were modest and metric dependent. The SSF-only configuration showed numerical improvement across all six evaluated metrics relative to the SwinUNETR baseline, suggesting that explicit integration of sweep-sequential and in-plane information may provide complementary information for pseudo-volumetric segmentation. In contrast, the addition of ACL produced smaller and less consistent changes, and the complete model did not consistently outperform the partial configurations. None of the prespecified module-level contrasts remained statistically significant after Holm correction. Accordingly, the proposed components should not be interpreted as providing uniformly additive performance gains; rather, they may affect different aspects of volumetric overlap, surface agreement, and structural continuity. The present ablation analysis was conducted at the module level, with SSF treated as an integrated methodological unit comprising complementary sweep-sequential modeling and feature recalibration. Accordingly, the analysis was not designed to isolate the individual contributions of the BiLSTM sweep-sequential branch and SE attention, and further component-level ablation studies will be needed to quantify their respective effects and potential interactions.

The subsequent clinical comparison study was not designed to determine which segmentation architecture performed best among the benchmark models. Rather, its purpose was to evaluate the potential clinical utility of the AI-driven 3D visualization framework developed in this study. Accordingly, visualizations generated using the proposed model were used for the clinical evaluation. In a clinical comparison study, this 3D visualization was associated with a consistent improvement in diagnostic accuracy across experts and fellows. Although the observed accuracy gains were not significant, the confidence-weighted accuracy scores for the overall examiners significantly improved. These findings suggest that 3D visualization may reduce diagnostic uncertainty and improve examiner confidence during fetal cardiac screening. These benefits may be particularly valuable in routine screening settings, where confidence in identifying normal and abnormal cardiovascular anatomy can influence referral decisions and examination efficiencies. 3D visualization may be useful for identifying abnormal findings and confirming normal cardiovascular anatomy. In fetal cardiac screening, the correct identification of normal fetuses is as important as the detection of abnormalities. The proposed 3D visualization workflow does not aim to replace physician judgment but rather supports clinical decision-making by providing an additional anatomical perspective. Therefore, it may benefit both expert and less experienced examiners.

### Limitations

This study has some limitations. First, the pseudo-3D volumes were reconstructed by stacking sequential frames acquired during a freehand sweep without external tracking of probe position. Consequently, inter-frame spacing along the sweep axis was not calibrated to physical distance, and variations in probe velocity could introduce geometric distortion along this axis. In addition, because the acquisitions were performed without cardiac synchronization or ECG gating, adjacent frames may correspond to different phases of the cardiac cycle. Thus, apparent inter-slice variation or discontinuity may reflect not only probe displacement but also cardiac motion. Accordingly, the reconstructed volumes should be interpreted as pseudo-3D anatomical representations rather than metrically or geometrically calibrated 3D volumes. Future studies incorporating external probe tracking and cardiac-gated acquisition will be necessary to disentangle spatial variation from cardiac motion and to quantitatively assess geometric accuracy. Second, this was a retrospective single-center study, and the generalizability of the visualization workflow to other institutions, patient populations, and ultrasound systems remains uncertain. To address the domain-shift effects, further validation using datasets obtained from ultrasound diagnostic equipment from other vendors at multiple centers remains warranted. Third, the segmentation test set comprised 30 normal fetuses, and the clinical comparison study included 14 normal fetuses and 6 fetuses with CHD, limiting the statistical power of the analyses. Further evaluation using independent external datasets is warranted to confirm the robustness of the workflow. Fourth, the reconstructed structures were limited to the major left- and right-sided circulatory pathways. Although this simplified representation was designed to support an intuitive understanding of global cardiovascular anatomy, it does not provide a detailed segmentation of each cardiac chamber and great vessel. The present CHD cohort was small and heterogeneous and was intentionally limited to cases involving cardiovascular anatomical abnormalities. Therefore, the findings should not be generalized to CHD as a whole or interpreted as evidence of diagnostic performance for any specific CHD subtype. Additional studies are warranted before applying this approach to a broader range of complex CHDs. The anomaly score also depends on the number of highest-anomaly voxels included in its calculation. In the sensitivity analysis, the mean ROC AUC across the five random-seed runs remained at or above 0.811 for all evaluated top-k settings, although individual seed-level AUCs showed modest variability, ranging from 0.783 to 0.867. These seed-level variations reflect stochastic variability in the diffusion-based inference procedure and should not be interpreted as estimates of subject-level sampling uncertainty. The optimal top-k value was not established in this exploratory study. Before clinical implementation, the anomaly-score definition, including the top-k parameter, should be optimized using an independent development cohort and subsequently evaluated in an external validation cohort, rather than selected on the basis of performance in the present test dataset. Finally, a clinical comparison study was conducted using stored ultrasound videos and reconstructed images, and the clinical impact of this workflow in real-time screening settings was not evaluated. A prospective clinical study should be conducted to evaluate the clinical usefulness of our AI-driven 3D cardiovascular visualization approach. Accordingly, the present findings should be interpreted as preliminary evidence of potential clinical utility rather than as evidence of readiness for clinical deployment.

## 5. Conclusions

In this study, we developed and evaluated an AI-driven 3D visualization of right- and left-sided fetal cardiovascular structures from 2D ultrasound sweep videos. This approach showed favorable segmentation performance for generating 3D anatomical representations and was associated with an improved confidence-weighted accuracy score in a clinical comparison study. By presenting 3D continuous circulatory pathways, this workflow provides an additional anatomical perspective that supports the spatial understanding of fetal cardiovascular anatomy during prenatal screening. The findings of this study suggest that our AI-driven 3D cardiovascular visualization supports clinical interpretation.

## Figures and Tables

**Table 1:** Performance comparison among 3D segmentation models.

Models	Year	mDice	msDice	mHD95	mclDice	mCC Error	mSC Error
SegResNet	2018	0.774	0.851	5.511	0.844	2.129	0.139
SwinUNETR-v2	2023	0.782	0.855	5.326	0.854	1.983	0.123
MedNeXt	2023	0.784	0.860	5.248	0.857	1.637	0.125
SwinMM	2023	0.747	0.741	7.730	0.810	2.546	0.146
3DINO	2025	0.760	0.822	4.661	0.827	2.679	0.138
Ours	2026	0.785	0.859	5.378	0.855	1.892	0.132

mDice, mean Dice coefficient; msDice, mean surface Dice coefficient; mHD95, mean 95th-percentile Hausdorff distance; mclDice, mean centerline Dice coefficient; mCC error, mean connected-components absolute error; mSC error, mean skeleton-continuity absolute error; 3D, three-dimensional.

**Table 2:** Comparison of the screening accuracy and confidence-weighted accuracy scores with and without the AI-driven 3D visual support.

Examiners	Accuracy		Confidence-Weighted Accuracy Score		
	Video Only	Video + 3D	Video Only	Video + 3D	*p* Value
Overall (*n* = 12)	0.779	0.808	6.525	6.925	0.002
Experts (*n* = 5)	0.820	0.830	6.770	7.210	0.063
Fellows (*n* = 7)	0.750	0.793	6.350	6.721	0.031

AI, artificial intelligence; 3D, three-dimensional.

## Data Availability

The datasets generated and/or analyzed during the current study are not publicly available in order to protect participant privacy.

## Supplementary Materials

The following supporting information can be downloaded at: https://www.mdpi.com/article/10.3390/bioengineering13090986/s1, Figure S1: Schematic overview of the DDPM-based anatomical plausibility assessment; Figure S2: ROC curves for distinguishing normal and CHD cases; Table S1: Fetus-level paired statistical comparisons between the proposed model and comparator models using five-model ensemble predictions; Table S2: Fold-specific single-model and five-model ensemble performance on the independent held-out test set; Table S3: Module-level ablation analysis of the sweep–spatial fusion architecture and anatomy-aware contrastive loss; Table S4: Robustness and sensitivity analyses of the DDPM-based anatomical plausibility assessment across random seeds and top-k anomaly-score settings; Table S5: Examiner-level statistical analysis of the clinical comparison study.

## Abbreviations

The following abbreviations are used in this manuscript:

AI	Artificial intelligence
AUC	Area under the curve
CHD	Congenital heart disease
CoA	Coarctation of the aorta
DORV	Double-outlet right ventricle
mDice	Mean values of the Dice coefficient
msDice	Mean values of the surface Dice
mHD95	Mean values of the 95% Hausdorff distance
ROC	Receiver operating characteristic
STIC	Spatiotemporal image correlation
TAPVC	Total anomalous pulmonary venous connection
TGA	Transposition of the great arteries
VSD	Ventricular septal defect
2D	Two-dimensional
3D	Three-dimensional
3VTV	Three-vessel trachea view
3VV	Three-vessel view
4CV	Four-chamber view
