# Supplementary material for: AI-Driven Three-Dimensional Reconstruction of Fetal Cardiovascular Pathways from Ultrasound Sweep Videos for Congenital Heart Disease Screening
Source: Bioengineering (Basel). 2026 Aug 26;13(9):986. doi: 10.3390/bioengineering13090986 (PMC13603691; doi:10.3390/bioengineering13090986)
Supplement: Supplementary file 1 [file bioengineering-13-00986-s001.zip › bioengineering-4503654-supplementary.pdf]

Three-Dimensional Visualization of the Cardiovascular Structures in Fetal Cardiac Ultrasound Videos Using Artificial Intelligence

Naoaki Harada, Masaaki Komatsu, Naoki Teraya, Shohei Wakisaka, Katsuji Takeda, Takashi Natsume, Tomonori Taniguchi, Nobuji Kouno, Satoshi Takahashi, Ken Asada, Syuzo Kaneko, Mayumi Kaneko, Reina Komatsu, Kazuki Iwamoto, Ryu Matsuoka, Akihiko Sekizawa and Ryuji Hamamoto.

The file contains
The legends of Supplementary Figures S1-2 and Table S1-5

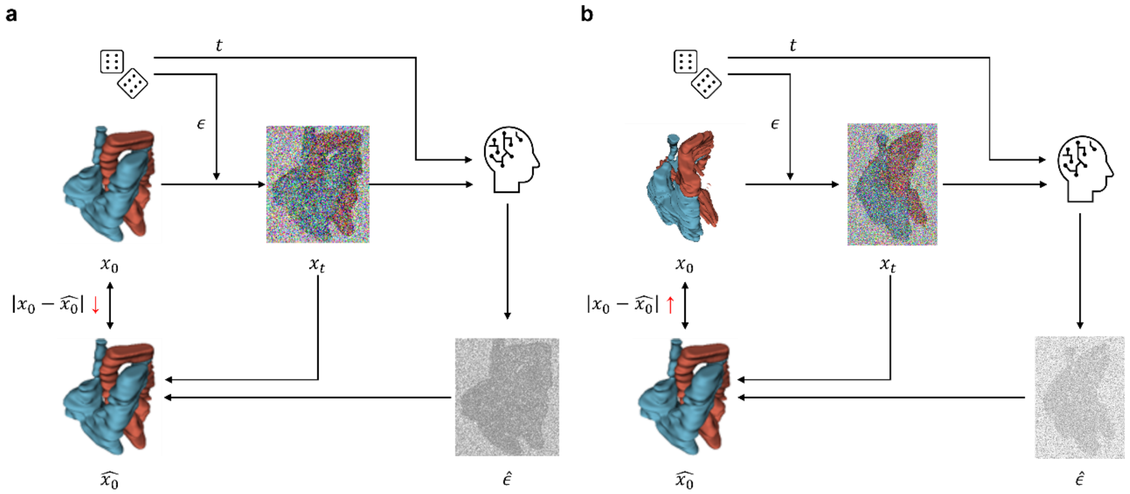

Supplementary Figure S1. Schematic overview of the DDPM-based anatomical plausibility assessment. For a predicted 3D cardiovascular volume with anatomically normal morphology, Gaussian noise ε was added at a fixed diffusion timestep t to generate the noisy volume x_t. The trained DDPM estimated the added noise and reconstructed the original volume $\hat{x}_0$. Because the input morphology was consistent with the normal anatomical distribution learned by the DDPM, the voxel-wise reconstruction error $|x_0 - \widehat{x_0}|$ was expected to remain small (**a**). The same reconstruction procedure was applied to a predicted volume containing morphology that deviated from the learned distribution of normal fetal cardiovascular anatomy. Such structures were expected to be reconstructed less accurately, resulting in a larger voxel-wise reconstruction error (**b**). The procedure was repeated using different Gaussian noise realizations, and the resulting reconstruction-error maps were averaged to obtain the final anomaly map. DDPM, denoising diffusion probabilistic model.

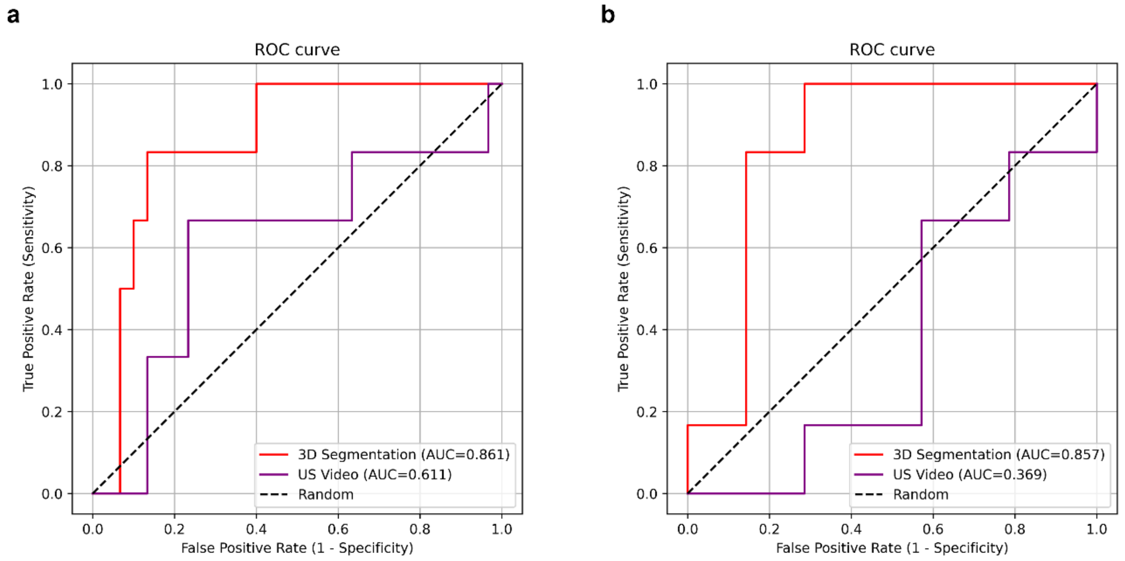

Supplementary Figure S2. ROC curves for distinguishing normal and CHD cases. Thirty normal cases in the test data for evaluating the 3D segmentation models (**a**) and 14 normal cases in the clinical comparison study cohort (**b**) were used to distinguish them from six CHD cases using anomaly scores derived from diffusion-based anatomical plausibility analysis. In both datasets, the diffusion model trained on AI-generated 3D cardiovascular reconstructions outperformed the model trained directly on fetal ultrasound sweep videos.

Supplementary Table S1. Fetus-level paired statistical comparisons between the proposed model and comparator models using five-model ensemble predictions.

Metric	Comparator	Ours (95% CI)	Comparator (95% CI)	Paired difference, Ours – comparator (95% CI)	Wilcoxon p	Holm-adjusted p	Rank-biserial effect size
mDice	SwinUNETR-v2	0.785 (0.768–0.802)	0.782 (0.767–0.797)	+0.003 (−0.005 to +0.010)	0.124	0.248	+0.325
	MedNeXt		0.784 (0.766–0.799)	+0.002 (−0.004 to +0.006)	0.177	0.248	+0.286
	SegResNet		0.774 (0.757–0.791)	+0.011 (+0.005 to +0.018)	0.003	0.009	+0.604
	SwinMM		0.747 (0.722–0.770)	+0.039 (+0.022 to +0.058)	<0.001	<0.001	+0.794
	3DINO		0.760 (0.739–0.779)	+0.026 (+0.015 to +0.038)	<0.001	<0.001	+0.837
msDice	SwinUNETR-v2	0.859 (0.834–0.881)	0.855 (0.836–0.874)	+0.003 (−0.005 to +0.011)	0.058	0.173	+0.398
	MedNeXt		0.860 (0.837–0.880)	−0.001 (−0.008 to +0.005)	0.556	0.556	+0.127
	SegResNet		0.851 (0.828–0.873)	+0.008 (+0.000 to +0.015)	0.109	0.219	+0.338
	SwinMM		0.741 (0.708–0.770)	+0.117 (+0.099 to +0.137)	<0.001	<0.001	+1.000
	3DINO		0.822 (0.794–0.847)	+0.037 (+0.026 to +0.050)	<0.001	<0.001	+0.948
mHD95	SwinUNETR-v2	5.378 (4.510–6.465)	5.326 (4.597–6.203)	+0.051 (−0.319 to +0.451)	0.777	1.000	−0.062
	MedNeXt		5.248 (4.460–6.185)	+0.130 (−0.321 to +0.593)	0.665	1.000	+0.095
	SegResNet		5.511 (4.702–6.444)	−0.133 (−0.521 to +0.284)	0.309	0.926	−0.217
	SwinMM		7.730 (6.150–9.681)	−2.352	<0.001	0.003	−0.695

				(−4.155 to −1.003)			
	3DINO		4.661 (3.815–5.710)	+0.717 (−0.223 to +1.520)	0.003	0.014	+0.596
mclDice	SwinUNETR-v2	0.855 (0.830–0.874)	0.854 (0.837–0.870)	+0.000 (−0.009 to +0.009)	0.808	1.000	+0.054
	MedNeXt		0.857 (0.833–0.875)	−0.002 (−0.011 to +0.005)	0.598	1.000	−0.114
	SegResNet		0.844 (0.823–0.863)	+0.010 (+0.000 to +0.020)	0.067	0.200	+0.385
	SwinMM		0.810 (0.781–0.836)	+0.045 (+0.023 to +0.069)	0.001	0.005	+0.647
	3DINO		0.827 (0.803–0.848)	+0.027 (+0.013 to +0.041)	<0.001	0.004	+0.673
mCC error	SwinUNETR-v2	1.892 (1.483–2.350)	1.983 (1.667–2.308)	−0.092 (−0.525 to +0.359)	0.522	0.629	−0.140
	MedNeXt		1.637 (1.288–2.042)	+0.254 (−0.033 to +0.562)	0.210	0.629	+0.297
	SegResNet		2.129 (1.583–2.808)	−0.237 (−0.763 to +0.250)	0.312	0.629	−0.220
	SwinMM		2.546 (1.950–3.183)	−0.654 (−1.125 to −0.158)	0.021	0.082	−0.519
	3DINO		2.679 (2.042–3.471)	−0.787 (−1.417 to −0.175)	0.010	0.049	−0.578
mSC error	SwinUNETR-v2	0.132 (0.109–0.157)	0.123 (0.101–0.146)	+0.009 (−0.004 to +0.023)	0.229	1.000	+0.256
	MedNeXt		0.125 (0.100–0.150)	+0.008 (−0.005 to +0.020)	0.265	1.000	+0.237
	SegResNet		0.139 (0.106–0.177)	−0.007 (−0.034 to +0.018)	0.952	1.000	−0.015
	SwinMM		0.146 (0.112–0.183)	−0.014 (−0.039 to +0.011)	0.371	1.000	−0.191

	3DINO	0.138 (0.110–0.166)	−0.005 (−0.026 to +0.016)	0.700	1.000	−0.084

Supplementary Table S2. Fold-specific single-model and five-model ensemble performance on the independent held-out test set.

Model	Prediction	mDice	msDice	mHD95	mclDice	mCC error	mSC error
Ours	Fold 1	0.769 (0.755–0.783)	0.846 (0.825–0.865)	5.640 (4.856–6.557)	0.831 (0.811–0.849)	2.812 (2.171–3.604)	0.130 (0.105–0.157)
	Fold 2	0.771 (0.752–0.788)	0.844 (0.820–0.865)	5.308 (4.555–6.174)	0.829 (0.803–0.850)	3.858 (2.925–4.925)	0.128 (0.098–0.161)
	Fold 3	0.766 (0.749–0.783)	0.840 (0.816–0.861)	5.969 (5.076–7.061)	0.824 (0.800–0.845)	3.608 (2.775–4.475)	0.131 (0.107–0.154)
	Fold 4	0.766 (0.746–0.785)	0.839 (0.812–0.862)	5.779 (4.739–7.214)	0.829 (0.800–0.853)	2.800 (2.167–3.533)	0.123 (0.102–0.147)
	Fold 5	0.770 (0.752–0.786)	0.846 (0.822–0.867)	5.273 (4.463–6.326)	0.831 (0.804–0.854)	3.862 (2.812–5.104)	0.123 (0.096–0.152)
	Mean ± SD across folds	0.769 ± 0.002	0.843 ± 0.003	5.594 ± 0.301	0.829 ± 0.003	3.388 ± 0.541	0.127 ± 0.004
	5-model ensemble	0.78 (0.768–0.802)	0.859 (0.834–0.881)	5.378 (4.510–6.465)	0.855 (0.830–0.874)	1.892 (1.483–2.350)	0.132 (0.109–0.157)
SwinUNETR-v2	Fold 1	0.760 (0.741–0.779)	0.832 (0.808–0.854)	6.159 (5.168–7.289)	0.822 (0.800–0.842)	3.179 (2.617–3.829)	0.121 (0.096–0.148)
	Fold 2	0.768 (0.751–0.785)	0.840 (0.818–0.862)	5.846 (5.046–6.817)	0.831 (0.807–0.851)	2.833 (2.158–3.742)	0.129 (0.107–0.153)
	Fold 3	0.761 (0.743–0.779)	0.836 (0.815–0.856)	5.858 (5.048–6.807)	0.825 (0.806–0.844)	3.146 (2.633–3.712)	0.127 (0.094–0.164)
	Fold 4	0.767 (0.751–0.782)	0.836 (0.817–0.854)	5.910 (5.281–6.657)	0.828 (0.809–0.846)	2.688 (2.138–3.283)	0.128 (0.100–0.161)
	Fold 5	0.770 (0.753–0.787)	0.843 (0.822–0.863)	5.935 (5.029–6.960)	0.835 (0.818–0.852)	2.625 (1.950–3.433)	0.113 (0.088–0.139)
	Mean ± SD across folds	0.765 ± 0.004	0.837 ± 0.004	5.941 ± 0.127	0.828 ± 0.005	2.894 ± 0.257	0.123 ± 0.007
	5-model ensemble	0.782 (0.767–0.797)	0.855 (0.836–0.874)	5.326 (4.597–6.203)	0.854 (0.837–0.870)	1.983 (1.667–2.308)	0.123 (0.101–0.146)
MedNeXt	Fold 1	0.765 (0.745–0.783)	0.843 (0.818–0.865)	5.491 (4.705–6.402)	0.833 (0.811–0.852)	2.475 (2.042–2.983)	0.127 (0.103–0.154)
	Fold 2	0.770	0.846	5.984	0.843	2.154	0.121

		(0.751–0.787)	(0.824–0.866)	(4.670–7.885)	(0.820–0.862)	(1.542–2.904)	(0.095–0.148)
	Fold 3	0.762 (0.744–0.780)	0.839 (0.815–0.861)	5.534 (4.752–6.395)	0.838 (0.817–0.856)	2.546 (1.992–3.104)	0.138 (0.108–0.171)
	Fold 4	0.768 (0.749–0.784)	0.841 (0.818–0.862)	5.855 (4.873–7.071)	0.839 (0.814–0.860)	2.129 (1.488–3.013)	0.124 (0.092–0.157)
	Fold 5	0.763 (0.742–0.781)	0.835 (0.808–0.858)	6.454 (5.309–7.757)	0.833 (0.811–0.853)	1.904 (1.413–2.479)	0.121 (0.094–0.149)
	Mean ± SD across folds	0.765 ± 0.003	0.841 ± 0.004	5.864 ± 0.391	0.837 ± 0.004	2.242 ± 0.265	0.126 ± 0.007
	5-model ensemble	0.784 (0.766–0.799)	0.860 (0.837–0.880)	5.248 (4.460–6.185)	0.857 (0.833–0.875)	1.637 (1.288–2.042)	0.125 (0.100–0.150)
SegResNet	Fold 1	0.760 (0.740–0.779)	0.837 (0.811–0.860)	6.594 (5.091–8.606)	0.829 (0.802–0.851)	2.542 (1.992–3.175)	0.129 (0.102–0.158)
	Fold 2	0.761 (0.739–0.780)	0.837 (0.807–0.863)	5.802 (4.892–6.886)	0.828 (0.800–0.852)	3.067 (2.283–4.142)	0.144 (0.117–0.172)
	Fold 3	0.759 (0.741–0.777)	0.836 (0.812–0.860)	6.312 (5.191–7.677)	0.826 (0.802–0.846)	3.154 (2.529–3.925)	0.129 (0.098–0.164)
	Fold 4	0.757 (0.737–0.775)	0.833 (0.808–0.856)	6.095 (5.188–7.177)	0.823 (0.798–0.845)	3.013 (2.271–3.971)	0.142 (0.108–0.179)
	Fold 5	0.758 (0.740–0.776)	0.835 (0.812–0.856)	5.919 (5.124–6.815)	0.829 (0.809–0.847)	2.587 (1.800–3.621)	0.136 (0.105–0.169)
	Mean ± SD across folds	0.759 ± 0.002	0.836 ± 0.002	6.144 ± 0.317	0.827 ± 0.002	2.872 ± 0.286	0.136 ± 0.007
	5-model ensemble	0.774 (0.757–0.791)	0.851 (0.828–0.873)	5.511 (4.702–6.444)	0.844 (0.823–0.863)	2.129 (1.583–2.808)	0.139 (0.106–0.177)
SwinMM	Fold 1	0.723 (0.696–0.749)	0.713 (0.679–0.744)	8.933 (6.966–11.239)	0.784 (0.752–0.815)	3.808 (2.975–4.700)	0.160 (0.123–0.198)
	Fold 2	0.737 (0.709–0.761)	0.730 (0.696–0.760)	7.654 (6.177–9.539)	0.798 (0.767–0.826)	3.104 (2.242–4.046)	0.147 (0.119–0.178)
	Fold 3	0.728 (0.700–0.754)	0.717 (0.683–0.748)	8.344 (6.553–10.467)	0.785 (0.753–0.816)	4.579 (3.646–5.617)	0.152 (0.118–0.188)
	Fold 4	0.723 (0.691–0.751)	0.717 (0.681–0.750)	8.599 (6.809–10.688)	0.775 (0.740–0.806)	4.104 (3.154–5.100)	0.167 (0.130–0.207)

	Fold 5	0.737 (0.714–0.759)	0.732 (0.702–0.760)	7.847 (6.308–9.825)	0.799 (0.772–0.826)	4.188 (2.975–5.625)	0.143 (0.108–0.182)
	Mean ± SD across folds	0.729 ± 0.007	0.722 ± 0.009	8.275 ± 0.527	0.788 ± 0.011	3.957 ± 0.550	0.154 ± 0.009
	5-model ensemble	0.747 (0.722–0.770)	0.741 (0.708–0.770)	7.730 (6.150–9.681)	0.810 (0.781–0.836)	2.546 (1.950–3.183)	0.146 (0.112–0.183)
3DINO	Fold 1	0.744 (0.724–0.764)	0.806 (0.779–0.829)	6.015 (4.249–8.924)	0.801 (0.775–0.825)	5.062 (3.913–6.396)	0.138 (0.105–0.173)
	Fold 2	0.733 (0.708–0.755)	0.790 (0.756–0.818)	5.295 (4.305–6.438)	0.792 (0.762–0.818)	4.467 (3.558–5.467)	0.153 (0.121–0.188)
	Fold 3	0.731 (0.707–0.753)	0.790 (0.760–0.816)	5.360 (4.576–6.321)	0.787 (0.762–0.810)	3.763 (2.929–4.708)	0.158 (0.125–0.195)
	Fold 4	0.741 (0.716–0.764)	0.803 (0.771–0.831)	5.114 (4.116–6.275)	0.801 (0.771–0.828)	3.808 (3.083–4.567)	0.148 (0.117–0.182)
	Fold 5	0.748 (0.727–0.766)	0.807 (0.781–0.831)	5.480 (4.331–6.892)	0.808 (0.784–0.829)	3.342 (2.658–4.075)	0.142 (0.107–0.180)
	Mean ± SD across folds	0.739 ± 0.007	0.799 ± 0.009	5.453 ± 0.341	0.798 ± 0.008	4.088 ± 0.677	0.148 ± 0.008
	5-model ensemble	0.760 (0.739–0.779)	0.822 (0.794–0.847)	4.661 (3.815–5.710)	0.827 (0.803–0.848)	2.679 (2.042–3.471)	0.138 (0.110–0.166)

Supplementary Table S3. Module-level ablation analysis of the sweep–spatial fusion architecture and anatomy-aware contrastive loss.

Model	SSF	ACL	mDice	msDice	mHD95	mclDice	mCC error
SwinUNETR-v2	No	No	0.782 (0.767–0.797)	0.855 (0.835–0.874)	5.326 (4.601–6.233)	0.854 (0.836–0.870)	1.983 (1.667–2.317)
SwinUNETR-v2 + ACL	No	Yes	0.785 (0.771–0.799)	0.860 (0.841–0.877)	5.289 (4.518–6.262)	0.854 (0.835–0.870)	2.058 (1.683–2.442)
SwinUNETR-v2 + SSF	Yes	No	0.786 (0.770–0.801)	0.859 (0.837–0.878)	5.320 (4.510–6.336)	0.857 (0.837–0.873)	1.904 (1.467–2.413)
SwinUNETR-v2 + ACL + SSF (Ours)	Yes	Yes	0.785 (0.767–0.801)	0.859 (0.834–0.880)	5.378 (4.506–6.481)	0.855 (0.830–0.874)	1.892 (1.483–2.342)

ACL, anatomy-aware contrastive loss; SSF, sweep–spatial fusion.

Supplementary Table S4. Robustness and sensitivity analyses of the DDPM-based anatomical plausibility assessment across random seeds and top-k anomaly-score settings.

Top-k voxels	ROC AUC (95% CI) by random seed					Mean ± SD across seeds
	Seed 300	Seed 4,810	Seed 9,930	Seed 10,430	Seed 10,980	
1	0.844 (0.680–0.969)	0.789 (0.609–0.933)	0.806 (0.626–0.942)	0.794 (0.634–0.929)	0.822 (0.661–0.945)	0.811 ± 0.023
5	0.861 (0.695–0.980)	0.794 (0.616–0.938)	0.811 (0.638–0.943)	0.794 (0.634–0.929)	0.822 (0.661–0.945)	0.817 ± 0.027
10	0.861 (0.695–0.980)	0.789 (0.609–0.935)	0.811 (0.638–0.943)	0.794 (0.633–0.930)	0.817 (0.652–0.943)	0.814 ± 0.028
20	0.861 (0.694–0.980)	0.806 (0.629–0.942)	0.811 (0.638–0.943)	0.789 (0.625–0.929)	0.828 (0.664–0.955)	0.819 ± 0.027
50	0.861 (0.691–0.980)	0.833 (0.661–0.966)	0.828 (0.661–0.961)	0.783 (0.611–0.926)	0.828 (0.657–0.961)	0.827 ± 0.028
100	0.867 (0.706–0.980)	0.856 (0.695–0.970)	0.844 (0.687–0.961)	0.789 (0.606–0.935)	0.833 (0.665–0.967)	0.838 ± 0.030
Mean ± SD across tested top-k settings	0.859 ± 0.008	0.811 ± 0.027	0.819 ± 0.015	0.791 ± 0.005	0.825 ± 0.006	—

Supplementary Table S5. Examiner-level statistical analysis of the clinical comparison study.

Examiners	Metric	Video Only, Mean (95% CI)	Video + 3D, Mean (95% CI)	Paired Difference, Mean (95% CI)	*p* Value	Rank-Biserial Correlation
Overall (n = 12)	Accuracy	0.779 (0.704–0.842)	0.808 (0.767–0.846)	0.029 (−0.004 to 0.067)	0.146	0.607
	Confidence-weighted accuracy score	6.525 (6.071–6.925)	6.925 (6.475–7.329)	0.400 (0.242–0.546)	0.002	0.949
Experts (n = 5)	Accuracy	0.820 (0.710–0.910)	0.830 (0.790–0.870)	0.010 (−0.040 to 0.080)	1.000	0.000
	Confidence-weighted accuracy score	6.770 (6.120–7.290)	7.210 (6.670–7.670)	0.440 (0.190–0.690)	0.063	1.000
Fellows (n = 7)	Accuracy	0.750 (0.657–0.821)	0.793 (0.736–0.843)	0.043 (0.014–0.079)	0.066	1.000
	Confidence-weighted accuracy score	6.350 (5.750–6.864)	6.721 (6.100–7.286)	0.371 (0.179–0.550)	0.031	0.929
